# Will the Use of Pharmacogenetics Improve Treatment Efficiency in COVID-19?

**DOI:** 10.3390/ph15060739

**Published:** 2022-06-13

**Authors:** Beata Franczyk, Jacek Rysz, Jarosław Miłoński, Tomasz Konecki, Magdalena Rysz-Górzyńska, Anna Gluba-Brzózka

**Affiliations:** 1 Department of Nephrology, Hypertension and Family Medicine, Medical University of Lodz, 90-549 Lodz, Poland; bfranczyk-skora@wp.pl (B.F.); jacek.rysz@umed.lodz.pl (J.R.); 2 Department of Otolaryngology, Laryngological Oncology, Audiology and Phoniatrics, Medical University of Lodz, 90-549 Lodz, Poland; jaroslaw.milonski@umed.lodz.pl; 3 Department of Urology, Medical University of Lodz, 90-549 Lodz, Poland; tomasz.konecki@umed.lodz.pl; 4 Department of Ophthalmology and Visual Rehabilitation, Medical University of Lodz, 90-549 Lodz, Poland; mrs-89@o2.pl

**Keywords:** COVID-19, treatment, pharmacogenetics

## Abstract

The COVID-19 pandemic is associated with a global health crisis and the greatest challenge for scientists and doctors. The virus causes severe acute respiratory syndrome with an outcome that is fatal in more vulnerable populations. Due to the need to find an efficient treatment in a short time, there were several drugs that were repurposed or repositioned for COVID-19. There are many types of available COVID-19 therapies, including antiviral agents (remdesivir, lopinavir/ritonavir, oseltamivir), antibiotics (azithromycin), antiparasitics (chloroquine, hydroxychloroquine, ivermectin), and corticosteroids (dexamethasone). A combination of antivirals with various mechanisms of action may be more efficient. However, the use of some of these medicines can be related to the occurrence of adverse effects. Some promising drug candidates have been found to be ineffective in clinical trials. The knowledge of pharmacogenetic issues, which translate into variability in drug conversion from prodrug into drug, metabolism as well as transport, could help to predict treatment efficiency and the occurrence of adverse effects in patients. However, many drugs used for the treatment of COVID-19 have not undergone pharmacogenetic studies, perhaps as a result of the lack of time.

## 1. Introduction

The sudden outbreak of the COVID-19 pandemic posed a great challenge to healthcare professionals and started a race with time to find appropriate pharmacological therapeutics [1]. However, since COVID-19 was killing a large number of people in a short period, the time to develop new drugs was very limited. The cause of COVID-19 is the severe acute respiratory syndrome coronavirus 2 (SARS-CoV-2) which is a complex disease that attacks several organ systems [2]. The coronavirus genome is made up of RNA [3]. Viruses with RNA genomes have RNA-dependent RNA polymerase that is highly conserved within species [4]. In order to enter the host, SARS-CoV2 binds primarily to the cell-membrane angiotensin-converting enzyme 2 (ACE2) receptors via the viral structural spike (S) protein [5]. Following the invasion, the virus activates cellular processes to synthesize viral proteins which enable the replication of the virus’s genetic material [6]. The SARS-CoV-2 disorder is usually manifested with mild-to-moderate symptoms of an upper respiratory tract infection, accompanied by myalgia and fatigue. However, sufferers can also develop severe pneumonia and even sudden development of respiratory failure [7]. Patients with severe illness (defined as SpO_2_ < 94%, PaO_2_/FiO_2_ < 300 mm Hg, respiratory rate > 30 breaths/min, or lung infiltrates > 50%) develop an uncontrolled immune response associated with an increased abundance of pro-inflammatory cytokines and chemokines, including IL1β, IL6, IL8, TNFα, MIP1α, and VEGF [8,9]. Such a “cytokine storm” is reported in patients with life-threatening complications requiring hospitalization and intensive care [10,11]. Apart from respiratory problems, patients with severe COVID-19 suffer also from serious complications including acute myocardial injury, renal failure kidney injury, abnormal liver function and liver failure, septic shock, blood leukocyte abnormalities, and disseminated intravascular coagulation [2,12]. The mortality rate of COVID-19 patients varies and depends on their clinical conditions. For example, male gender, age, the requirement for ICU care, obesity and concomitant chronic conditions, such as cardiovascular, oncologic, neurodegenerative and metabolic diseases are associated with higher mortality [13].

Drug discovery requires several years to proceed from the concept to availability in the market [14]. This task became even more complicated when the virus was found to mutate at a fast pace. Since many old drugs have shown promising therapeutic properties that can possibly be exploited in the treatment of COVID-19 patients, drug repurposing became a favorable option to obtain efficient therapeutics [11]. However, the development of a precise, standardized protocol for repurposing is necessary. Despite being less time-consuming, the repurposing still requires clinical trials to confirm the efficiency and safety in a different target group of patients. No definitive drugs or vaccines for the treatment or prevention of COVID-19 have been approved in 2021, available therapies only obtained ‘conditional approval’ [15]. Drug repurposing is a cost- and time-saving approach to managing COVID-19 [12,13,14]; however, limitations such as low success rate and the possibility of adverse side effects cannot be overlooked [13,14,15,16,17].

There are many types of available COVID-19 therapies, including antiparasitics (chloroquine, hydroxychloroquine, ivermectin), antiviral agents (remdesivir, lopinavir/ritonavir, oseltamivir), antibiotics (azithromycin), and corticosteroids (dexamethasone) [8,9]. A combination of antivirals with various mechanisms of action may be more efficient, however, their adverse effects should not be underrated [9,10]. They are mainly used to treat symptoms and their choice depends on disease severity [2]. Both the efficiency and recommendations concerning COVID-19 treatments remain controversial due to the lack of drug response or the incidence of adverse drug reactions (ADR) in some patients.

Pharmacogenetics is the field of science that could elucidate inter-individual variability of drug response on the basis of genetic alterations present in COVID-19 patients [16]. Genetic factors are responsible for at least part of the unpredictability of COVID-19 therapy outcome and side effects [17]. The identification and characterization of gene variants (especially in genes encoding drug-metabolizing enzymes, transporters, or receptors) which influence the pharmacokinetics and pharmacodynamics of medications used enable the choice of the most appropriate drug for the patient and help to avoid ADRs [2,18].

This review focuses on the pharmacogenetic aspects of drugs used in the therapy of COVID-19 in 2020–2021. Therefore, we selected those drugs (used in the years 2020–2021) to research the possible impact of any existing pharmacogenetic issues on efficiency or adverse effects. We used the following keywords during our search: COVID-19 drugs, repurposing, pharmacogenetics, and single nucleotide polymorphisms (SNP).

## 2. Remdesivir (GS-441524)

Remdesivir (GS-441524) is a monophosphoramidate nucleoside analog prodrug whose main purpose was to treat the Ebola virus disease [2]. This agent acts as an analog of adenosine triphosphate (ATP) which competes with the natural ATP substrate for the incorporation into nascent RNA chains by viral RNA-dependent RNA polymerase to hinder viral replication since its activity results in premature chain termination during replication of the viral RNA [19].

The results of studies conducted on animal models have revealed that this drug shows some efficacy in the treatment of coronavirus diseases, including SARS-CoV-2 and the Middle East Respiratory Syndrome coronavirus (MERS-CoV) [20,21]. Additionally, the data from clinical trials provided low-certainty evidence that remdesivir may decrease time to clinical improvement, the occurrence of severe adverse events, and reduce mortality of patients with severe COVID-19 [22,23]. A double-blind, randomized, placebo-controlled trial (NIAID ACTT-1 Study (CO-US-540-5776)) of intravenous remdesivir in hospitalized adults with mild/moderate or severe COVID-19 disease demonstrated the shortening of time to recovery in patients with severe symptoms, however, no differences were found in a group of patients with mild/moderate disease [24]. The best outcomes were reported in hospitalized individuals receiving oxygen on Day 1, however, not on ventilation. This medicine received conditional approval for the treatment of COVID-19. Remdesivir was the first drug authorized as an investigational therapeutic option for COVID-19 [25]. It is recommended as a single 200 mg dose, followed by a 100 mg daily infusion [26].

The application of pharmacogenetics has been suggested to improve the efficacy of such treatment and decrease its toxicity. Remdesivir, which is administered in the form of a prodrug, requires the conversion into active form—GS-443902 by esterases (which leads to the formation of intermediate metabolite, GS-704277) followed by phosphorylation. CYP2C8, CYP2D6, and CYP3A4 (to a lesser extent) enzymes were found to affect the metabolic conversion of remdesivir [27]. Numerous studies have demonstrated that enzymes of cytochrome P450 are very polymorphic. The Pharmacogene Variation Consortium have described over 140 identified CYP2D6 alleles differing in enzyme activity; some variants are lacking enzymatic activity (e.g., CYP2D6*3, *4, *6), and other display diminished activity (e.g., CYP2D6*10, *17, *29 or *41) or enhanced activity due to duplication or multiplication of active alleles (e.g., CYP2D6*1xN, *2xN) [28]. The prevalence of some CYP2D6 polymorphisms was found to be higher in some populations. So far, more than 34 allelic variants have been identified, however, there is no detailed clinical data concerning their significance [29]. No non-functional alleles of *CYP2C8* have been described yet [29]. Since cytokines released in the process of inflammatory responses (e.g., interleukin-6; IL-6) reduce the expression of CYP enzymes (i.e., CYP3A4), it appears that polymorphisms within the *CYP3A4* gene may affect the response to the treatment. The underlying mechanism for CYP down-regulation involves the translational activation of C/EBPbeta-LIP which competes with and counteracts constitutive C/EBP transactivators [30].

The knowledge of the presence of specific P450 alleles combinations helps to predict the metabolic phenotype of the patient. Individuals who have two functional alleles are classified as extensive metabolizers, those with duplicated or multiplied functional alleles display the ultrarapid metabolizers phenotype. In turn, individuals carrying two null alleles are poor metabolizers, while those with one functional allele and one null allele are intermediate metabolizers [31]. All these patients may require the modification of drug dose to provide optimal efficiency and avoid serious adverse events. Apart from CYP enzymes, remdesivir has been demonstrated to be a substrate for the organic anion transporting polypeptide 1B1 (OATP1B1) and P-glycoprotein (P-gp) [32]. The first one belongs to the transmembrane family of transport proteins and is responsible for the uptake of substances into the cells of various organs, primarily the liver, while the latter one is an efflux pump [33]. OATP1B1 transporter is encoded by *SLCO1B1* gene and until now several allele variations affecting drug disposition (rs2306283, rs56101265, rs56061388, rs72559745, rs4149056, rs72559746, rs55901008, rs59502379, and rs56199088) have been found. All these variants are associated with reduced transporter function [34]. In turn, P-gp is encoded by *ABCB1* gene (belonging to adenosine triphosphate (ATP)-binding cassette (ABC) genes) which is also polymorphic (e.g., rs1128503, rs2032582, and rs1045642). The results of studies have revealed that P-gp is involved in viral resistance and trafficking cytokines and enveloped viruses [35]. The aforementioned variants could theoretically affect the pharmacokinetics of remdesivir, but since the risk of significant genetic pharmacokinetic interactions seems to be low, no guidelines recommend pharmacogenetic testing before the administration of this drug [3]. No pharmacogenomic analysis has been performed so far. Ongoing clinical trials which study the antiviral activity of remdesivir in patients with COVID-19 include NCT04292899, NCT04292730, NCT04257656, NCT04252664, and NCT04280705 [36].

## 3. Lopinavir/Ritonavir

Lopinavir is a human immunodeficiency virus (HIV) protease inhibitor, which, in combination with ritonavir, can suppress viral replication [2]. It was demonstrated to block a post-entry step in the MERS-CoV replication cycle, thus it appears a potential agent for COVID-19 treatment [37]. A computational approach used to screen for available commercial medicines that may function as inhibitors for the protease M^pro^ of 2019-nCoV (key enzyme for coronavirus replication) has revealed that lopinavir and ritonavir are potential candidates [38]. The results of studies indicate that M^pro^ of different coronaviruses is highly conserved in terms of sequence and 3D structures and it is of high functional importance [39]. Therefore the M^pro^ is considered an attractive target for the design of anticoronaviral drugs [38]. Moreover, it has been found that lopinavir and ritonavir could act as inhibitors of SARS-CoV-2 3CLpro protein which cleaves polyproteins into an RNA-dependent RNA polymerase and a helicase 1 during the replication process. However, treatment with lopinavir and ritonavir is not currently recommended in the therapy of COVID-19 due to a lack of efficacy in a small randomized controlled trial [40].

Lopinavir undergoes rapid metabolism by the CYP3A4 enzyme and therefore the co-administration of a low dose of a powerful inhibitor of CYP3A4 (ritonavir) is necessary to achieve optimal plasma concentrations of lopinavir for its antiretroviral activity [41,42]. Moreover, ritonavir inhibits the P-gp in the gut wall thus ameliorating lopinavir absorption [43]. Standard regimen treatment with lopinavir/ritonavir includes the use of the following doses: 400 mg/100 mg bid for adults for 10–21 days [44]. Variants in *CYP3A5*, *ABCC2*, and *SLCO1B1* genes have been suggested to potentially affect plasma concentrations of lopinavir. However, conflicting results were obtained from various pharmacogenetic studies. Variants within *CYP3A4* and *ABCB1* genes can modulate the pharmacokinetics and pharmacodynamics of both lopinavir and ritonavir. Ritonavir is also metabolized by CYP2J2, CYP3A5, and CYP2D6 [3]. The fact that this drug increases concentrations of other medications metabolized by CYP3A4 should be taken into consideration if it is used as a polytherapy. Lopinavir and ritonavir are contraindicated with drugs that are highly metabolized by CYP3A enzymes or are potent CYP3A inducers [25]. Moreover, ritonavir may also affect the biotransformation of some medicines metabolized via UGT-catalysed glucuronidation [3].

The carriers of CYP3A4*22/*22, alone or in combination with *SLCO1B1* rs4149056 have been found to require a reduced dosage of lopinavir/ritonavir due to higher lopinavir trough concentration. In turn, the polymorphism rs28371759 CYP*18B within CYP3A4 (occurring more frequently in East Asians) is associated with enhanced CYP3A4 activity and thus faster metabolism of drugs like lopinavir [45]. Another variation that could affect the treatment with lopinavir and ritonavir is CYP3A5*3. The results of studies indicated that carriers of CYP3A5*3/*3 genotypes were lacking CYP3A5 expression as a result of altered mRNA splicing [46]. Therefore, it has been suggested that such patients may require lower doses of lopinavir and ritonavir.

Lopinavir and ritonavir are substrates of efflux transporter protein encoded by the ABCB1 gene. Approximately 66 coding SNPs have been identified within the ABCB1 gene [47]. The following polymorphisms: rs2032582 alleles T and A, rs9282564, and rs2229109 have been suggested to raise the drug concentration via diminishing the efflux of ABCB1. These SNPs occur in various populations with different frequencies. rs2032582 A allele is most prevalent in Africans (in 90% of the population), rs2032582 T allele in East Asians, and rs9282564 in Europeans. These patients may show a more pronounced response to the drugs transported by the ABCB1 [3]. The presence of rs8187710 variant in *ABCC2* (adenosine triphosphate (ATP)-binding cassette subfamily C member 2) encoding human canalicular multispecific organic anion transporter, also termed multidrug resistance-associated protein 2 (MRP2)), was associated with higher accumulation of lopinavir in peripheral blood mononuclear cells of HIV-treated patients [48,49]. In contrast, patients carrying the T allele of *ABCC2* rs717620, when compared to CC homozygotes, showed decreased estimated glomerular filtration rate [50]. In turn, the analysis of the impact of genetic variants on lopinavir/ritonavir-related toxicity in HIV patients revealed that polymorphisms within *CETP*, *MCP-1*, *ABCC2*, *LEP*, and *SLCO1B3* genes were associated with the incidence of dyslipidemia and hyperbilirubinemia, while the variant in IL-6 was related to the occurrence of diarrhea (all *p* < 0.01) [51].

Ritonavir was demonstrated to inhibit also organic anion transporting polypeptides (OATP1B1 and OATP1B3) [52]. The polymorphism rs4149056 in *SLCO1B1* was found to be associated with decreased OATP1B1 transport activity in vivo, variations of lopinavir plasma concentrations, and 37% lower clearance. A reduced clearance is associated with lower uptake by hepatocytes which, in turn, translates into elevated plasma concentrations of lopinavir in C allele homozygotes [53]. Pharmacogenomic analysis of lopinavir/ritonavir in HIV-infected Caucasians demonstrated higher clearance of lopinavir in carriers of SLCO1B1*4/*4 variation, and lower clearance in those who had two or more variant alleles of SLCO1B1*5, *ABCC2* or a *CYP3A* tag compared to the reference group (12.6 vs. 3.9 vs. 5.4 l/h, respectively, *p* < 0.01) [45].

## 4. Azithromycin

Azithromycin is an azalide, macrolide antimicrobial agent with anti-inflammatory properties which is used to treat patients with viral infections [2]. It prevents severe respiratory tract infections since it hinders bacterial protein synthesis via binding to the 50S component of the 70S ribosomal subunit [54]. The combination of azithromycin and hydroxychloroquine (HCQ) has been suggested to exert a synergistic effect on COVID-19 disease [55]. The effectiveness of these two drugs in the treatment of COVID has been suggested not to be associated with the prevention of infection, but with their lysosomotropic properties which buffer the acidic environment (pH 4–5) of the endolysosomal lumen where SARS-CoV-2 moves after ACE-2 receptor-mediated endocytosis [56]. The increase of intravesicular pH up to near 7.0 is associated with the impairment of lysosomal functions and activities responsible for virus entry and replication cycle [56,57]. In general, macrolide agents trigger many drug–drug interactions, however, azithromycin is not an important substrate of CYP3A4, SLCO1B1, or SLCO1B3, and therefore its administration is associated with fewer interactions compared to other macrolides [58]. Azithromycin does not interact with cytochrome P450 enzymes, therefore genetic variations within this group of enzymes cannot affect treatment with azithromycin. However, its pharmacokinetics can be influenced by the activity of the P-glycoprotein (P-gp) transporter encoded by ABCB1 [3,59]. Scherrmann JM [57] suggested that ABCB1 might act as an enhancer by extensively confining azithromycin when the trapping is solely dependent on the passive diffusion. The interaction of azithromycin with P-gp was suggested to be the reason for its efficacy in the COVID-19 treatment as well as its synergistic actions when co-administrated with hydroxychloroquine [55,56]. Polymorphisms within the ABCB1 were demonstrated to alter peak azithromycin concentrations in healthy volunteers after a single dose (500 mg) [60]. Marked differences in C_max_ and T_max_ of azithromycin were associated with the presence of the ABCB1 rs2032582/rs1045642 genotype. Increased systemic exposure to azithromycin, especially when it is combined with hydroxychloroquine or chloroquine, may be even life-threatening as a result of the additive effects of both drugs on QT prolongation which may cause fatal arrhythmias [40].

## 5. Chloroquine and Hydroxychloroquine

Chloroquine (CQ) and hydroxychloroquine (HCQ) are drugs used to treat and as prophylaxis for malaria and autoimmune diseases such as rheumatoid arthritis (RA) and systemic lupus erythematosus (SLE). Additionally, chloroquine has been used to treat amebiasis and hydroxychloroquine has been used to prevent thrombosis among patients with anti-phospholipid antibody syndrome [2,61,62]. According to studies, chloroquine and hydroxychloroquine inhibit the expressions of major histocompatibility complex (MHC) class II, limit the formation of IL-1, IFNα, and TNF, as well as impair the activity of cyclic GMP-AMP synthase partly via the accumulation of phagocytic cells in lysosomes and autophagosomes and altering local pH concentrations [63]. Chloroquine and hydroxychloroquine were among the most frequently administered repurposed therapeutic agents during the first stages of the pandemic [25]. They are the first antiviral drugs formally designated for COVID-19 treatment as FDA Emergency Use Authorization was issued on 28 March 2020 [3]. However, later on, new data which suggested that these drugs’ potential benefits may not outweigh their identified potential risks were published [63]. The clinical trials assessing the efficacy and safety of these drugs are still ongoing. In the treatment of COVID-19, CQ is administered orally at a dose of 300 mg (adults), twice a day for a maximum of 10 days, while in the case of HCQ, the loading dose of 400 mg twice a day during the first day and then 200 mg bid (10 days average) is used [3].

The results of studies indicated that CQ and HCQ were effective in the fight against SARS-CoV-2 [64]. These two drugs hamper virus entry by targeting the endosomal pathway. They are capable of increasing the pH of endosomes and inhibiting membrane fusion [17]. Moreover, both drugs inhibit the glycosylation of host receptors and proteolytic processing thus blocking viral entry [65]. The immunomodulatory effects of these drugs have been suggested to facilitate the management of the cytokine storm associated with advanced COVID-19 disease [40]. In addition, their impact on hampering autophagy/lysosomal activity has been described [65,66]. SARS-CoV-2 virus during the invasion of the host body uses ACE2 receptors expressed on host epithelial cells as well as cofactor transmembrane protease, serine 2 (TMPRSS2). It has been suggested that some variants in *ACE2* (e.g., rs762890235 and rs1316056737) may prevent the interaction with the S protein and affect the clinical efficacy of HCQ or CQ [3]. One of the studies revealed that the ACE2 variant rs41303171 may stimulate the activation of TMPRSS2 and facilitate subsequent viral entry [67]. It is possible that HCQ and CQ may only be effective in the treatment of TMPRSS2-deficient patients who are infected by SARS-CoV-2, while such treatment exerts lower or no effect in those with wild-type TMPRSS2 [3].

Both drugs are broadly metabolized in the liver by CYP2C8, CYP3A4, CYP3A5, and, to a lesser extent, CYP2D6 [2,68]. The results of studies indicated that polymorphisms within these genes could affect malaria treatment with chloroquine. Carriers of CYP2C8*4 (missense mutation) have lower enzyme activity which translates into lower treatment efficacy compared to wild-type allele *1 A [69]. In turn, the allele CYP2D6*10 was found to be associated with the hydroxychloroquine metabolic ratio [70]. Patients with systemic lupus erythematosus demonstrated a ~20% increased ratio of the active metabolite of hydroxychloroquine metabolism to its parent drug in individuals carrying variants in CYP2D6 (rs1135840: CC vs. GG was 0.90 vs. 0.69, respectively, *p* < 0.01) [70]. The authors suggested that the occurrence of CYP polymorphisms may explain the wide variation in this drug levels in the blood. In turn, individuals with the CYP2D6*4 (rs3892097) variant have hardly any or even no CYP2D6 activity as a result of a splicing defect in an intron (between the third and fourth exon) [15,71]. Hareedy et al. [15] hypothesized that desethyl HCQ could be primarily responsible for the actions of HCQ, therefore the rate of its formation via CYP2D6 and other CYP enzymes could modify COVID-19 status among HCQ receivers for rheumatoid arthritis. In carriers of CYP2D6*4 (AA), HCQ is poorly metabolized which results in reduced formation of desethyl HCQ, thus probably diminishing the efficiency of HCQ in preventing COVID-19 viruses from entering cells. Lower levels of desethyl HCQ in individuals with CYP2D6*4 (AA) were found to be associated with a significantly higher grade of ground-glass opacities in the chest CT compared to wild and heterozygous carriers (but the *p*-value was >0.05) [15]. Due to the fact that both CQ and HCQ were found to inhibit CYP2D6 activity, they may not only potentiate the effects of other CYP2D6 substrates, including carvedilol and labetalol but also reduce the effectiveness of prodrugs that are activated by CYP2D6 (codeine and tramadol) [72,73]. In addition, CYP2C8*2 (rs11572103) and CYP2C8*3 (rs11572080) display a markedly decreased activity in vitro [74]. The study including malaria-infected patients found that low-activity alleles of *CYP2C8* (i.e., *2, *3, and *4) were associated with a worse decrease in gametocytaemia than wild-type alleles one day after chloroquine/primaquine treatment (−2.21 vs. −11.18 gametocytes/μL, *p* = 0.007, respectively), while individuals with variant alleles of *SLCO1A2* and *SLCO1B1* had reduced gametocytaemia clearance compared to the wild-type alleles (*p* = 0.018 and 0.024, respectively) [69,75]. It confirms that variations within these drug transporters may be crucial in the pharmacogenetics of chloroquine [76]. Hydroxychloroquine and chloroquine are substrates of organic anion transporting polypeptides (OATP), influx cellular membrane transporters encoded by *SLCO14*. Since genes involved in the pharmacokinetics of these two drugs are considered “very important pharmacogenes (VIPs)” by the PharmGKB, the variations within them may markedly affect the outcome of treatment [40].

Patients with known glucose-6 phosphate dehydrogenase deficiency should receive a reduced dose due to the risk of hemolysis and hemolytic anaemia [77]. High individual and ethnical dissimilarities in G6PD activity are related to genetic variations within its gene. For example, the deficiency of enzymatic activity was found to be frequent in Europe, the Mediterranean area, the Middle East, Asia, as well as in some regions of Africa [78]. The results of studies have indicated that as much as 39% of COVID-19 patients suffering from lupus were intolerant to HCQ or failed to respond to the treatment [10,79]. The most severe complications associated with HCQ and CQ treatment include QTc prolongation and ventricular arrhythmias which can be highly dangerous, especially for critically ill patients [80]. HCQ has a better safety profile compared to chloroquine [15]. The results of a large retrospective study of hospitalized patients with COVID-19 conducted in the New York metropolitan region revealed that patients treated with hydroxychloroquine and azithromycin had a higher prevalence of cardiac arrest and possibly higher mortality [81]. Long-term chloroquine and hydroxychloroquine treatment was found to induce retinal toxicity, while hydroxychloroquine alone can cause drug-related retinopathy which endures even following the cessation of therapy, as well as ocular toxicity and visual disturbances (retinal and macular toxicity) [82,83]. Retinopathy occurs more frequently in case of prolonged, high-dose treatment with hydroxychloroquine and chloroquine [84].

## 6. Corticosteroids

The infection with SARS-CoV-1 and MERS is associated with an unregulated inflammatory response which could ultimately severely affect the respiratory system [25,85]. Since patients with severe COVID-19 develop inflammatory-induced lung injuries, it seems that corticosteroids, including dexamethasone hydrocortisone, methylprednisolone, prednisone, and prednisolone are a good treatment option in this group [86]. Corticosteroids can be administered to decrease lung inflammatory responses which in many patients evolve into acute lung injury and acute respiratory distress syndrome (ARDS) [10]. According to the results of previous studies, corticosteroid treatment seems not to increase 90-day mortality (adjusted odds ratio, 0.75; 95% confidence interval (Cl), 0.52–1.07; *p* = 0.12), however, it delays MERS coronavirus RNA clearance in the respiratory and blood tracts (adjusted hazard ratio (aHR) 0.35; 95% CI, 0.17–0.72; *p* = 0.005) [87].

The data obtained from combined two meta-analyses based on pooled data from 8 randomized trials (including 7184 participants) of the systemic use of corticosteroids for COVID-19 demonstrated that such treatment is plausible to decrease 28-day mortality in patients with critical COVID-19 (relative risk [RR] 0.80, 95% CI 0.70–0.91) and severe disease (RR 0.80, 95% CI 0.70–0.92) [88]. However, such therapy was found to possibly increase the risk of mortality when administered to patients with non-severe COVID-19 (RR 1.22, 95% CI 0.93–1.61). Moreover, systemic corticosteroid treatment may diminish the need for invasive mechanical ventilation (RR 0.74, 95% CI 0.59–0.93). Based on these results, the WHO issued recommendations, according to which systemic corticosteroid therapy (6 mg/day of dexamethasone orally or intravenously or 50 mg/day of hydrocortisone intravenously every 8 h) should be used for 7 to 10 days in patients with severe and critical COVID-19 (with strong evidence). Corticosteroid therapy should not be used in patients with non-severe COVID-19 [88]. Currently, the role of corticosteroids in the therapy of COVID-19-infected patients is primarily limited to those with ARDS [40]. Both low (25 mg/day) and moderate (140 mg/day) doses of corticosteroids were found to decrease mortality in patients with acquired pneumonia [89]. Unfortunately, the risk of excessive persistence of viral load, secondary infections as well as long-term complications while using high-dose corticosteroids cannot be excluded [10]. The response to corticosteroids as well as associated toxicities may be associated with the presence of some alleles variants in genes, including those involved in the receptor binding (e.g., *CRHR1*, *NR3C1*), metabolizing enzymes (e.g., *CYP3A4*, *CYP3A5*, *CYP3A7*, *GSTT1*), chaperone/cochaperone protein (e.g., *ST13*, *STIP1*, *FKBP5*) as well as transporters (e.g., *MDR1*, *ABCB1*) [90]. The metabolic and mechanistic pathways of steroids are complex, there is no sufficient evidence supporting the use of any of them to adjust the treatment of patients with COVID-19. The only variants associated with corticosteroids in PharmGKB had a level of evidence higher than “low” (level 3), however, they were evaluated in the case of combination therapy (e.g., with chemotherapy) or inhaled corticosteroids. Therefore, currently, no pharmacogenetic guideline has been published on any corticosteroid and no pharmacogenetic recommendations have been made [25].

Dexamethasone is one of the glucocorticosteroids used to suppress cytokine release and hinder lung infiltration by neutrophils and other leukocytes [2]. This drug is metabolized by hepatic CYP3A4 into 6-hydroxydexamethasone and other metabolites [91]. It is also a substrate of P-gp (encoded by the *ABCB1* gene) which appears to contribute to steroid resistance [92]. Polymorphisms within the ABCB1 gene, an efflux transporter participating in the transport of xenobiotics across numerous body compartments, have been demonstrated to be associated with variability of the effectiveness of dexamethasone, and the tolerability of prednisone, methylprednisolone, and prednisolone, probably as a result of differences in drug exposure in various diseases [25,93,94]. In PharmGKB, there are some variants that were found to affect the response and/or the toxicity to dexamethasones, such as ABCB1 and others. However, due to the fact that analyses were performed on patients treated with different drugs, the obtained results should be treated with caution. In turn, a study searching for pharmacogenetic loci with the use of >300 expression microarrays from lymphoblastoid cell lines in dexamethasone-treated and untreated cells derived from asthmatic subjects has revealed new important variant sites, including 17 G/A rs6504666 and rs1380657 (*SPATA20*), rs12891009 (*ACOT4*), rs2037925 and rs2836987 (*BRWD1*), rs1144764 (*ALG8*), and rs3793371 (*NAPRT1*) [95].

## 7. Atazanavir (ATV)

Atazanavir is an established protease inhibitor used for the treatment of AIDS, with good oral bioavailability, efficacy, and safety profiles [3,10]. Usually, this drug is administered in combination with ritonavir or other antiviral drugs in highly active antiretroviral therapy despite the fact that concomitant use with ritonavir reduces the efficacy of atazanavir [25,96]. When compared to other protease inhibitors, atazanavir is less expected to cause lipodystrophy [10]. The results of in vitro and ex silico studies have suggested that atazanavir can mediate SARS-CoV-2 major protease inhibition [25,97,98]. Atazanavir repressed SARS-CoV2 replication in human epithelial pulmonary cells (A549) [3]. This drug has been found to be metabolized by CYP3A and act as an inhibitor of CYP3A and UGT1A. The results of pharmacogenetic studies carried out on HIV patients suggest the impact of polymorphisms within *UGT1A1*, *CYP3A4*, *CYP3A5*, and *SLCO3A1* genes on the efficacy of atazanavir [10]. The Clinical Pharmacogenetics Implementation Consortium (CPIC) guideline concerning this drug mentions several polymorphisms in *UGT1A1*, such as a variable dinucleotide (TA) repeat within the gene promoter region (rs8175347, alleles UGT1A1*28, *36 and *37) and the SNPs rs4148323 (UGT1A1*6) and rs887829 (UGT1A1*80) [3,99]. Carriers of TA repeats within the *UGT1A1* gene promoter region can be normal metabolizers (NMs), intermediate metabolizers (IMs), or poor metabolizers (PMs). Individuals with one or two copies of the *1 allele may metabolize atazanavir more quickly than individuals with one or more copies of the *3, *6, or *7 alleles [10,100]. Variants within *UGT1A1*, *UGT1A7*, *UGT1A3*, *APOE*, and *APOC3* genes have been suggested to be also associated with toxicity and ADRs [50,101,102]. Individuals carrying two alleles with decreased function (PMs) were found to be more likely to develop jaundice. In such patients, alternative therapy should be considered as side effects may cause nonadherence [25,99]. Moreover, homozygous carriers of the UGT1A1*28 allele appear to be predisposed to severe hyperbilirubinemia [103]; this risk is intermediate in UGT1A1*28 heterozygotes [104]. For carriers of UGT1A1*28/*28, *28/*37, *37/*37, and −364 TT, the CPIC guidelines recommend counseling on the possibility of developing hyperbilirubinemia before the initiation of the therapy [99]. *UGT1A1* genotyping performed in COVID-19 patients who are treated with atazanavir could improve therapy tolerability [3]. Atazanavir is also metabolized (to a lesser extent) by the P-gp efflux pump encoded by the multidrug resistance 1 (*MDR1*) gene. Homozygotic carriers of rs1045642 CC alleles, which appear to raise plasma levels of this drug, are at increased risk of hyperbilirubinemia and severe jaundice. Finally, *ABCA1* (rs542671182), *APOC3* (rs2070668, rs2854116, rs2854117), *APOA5* (rs 662799 and rs3135506), and *APOE* (ε2 and ε3 haplotypes) gene polymorphisms increase the risk of dyslipidaemia [105].

## 8. Tocilizumab

Since severe COVID-19 is associated with a cytokine-release syndrome with increased interleukin-6 (IL-6), it seems reasonable to use monoclonal antibodies directed against key inflammatory cytokines as the treatment option [40]. Interleukin-6 (IL-6) is a key trigger of this dysregulated inflammation [106]. It appears that monoclonal antibodies against IL-6 can reduce this process and ameliorate clinical outcomes [10]. One of the IL-6 receptor inhibitors—tocilizumab—is commonly used in the treatment of rheumatoid arthritis (RA) and cytokine-release syndrome triggered by chimeric antigen receptor-T cell therapy [40]. Due to its potential, it is currently under investigation for COVID-19. Typical doses of tocilizumab are 400 mg or 8 mg/kg in one or two doses, with the second dose given 8–12 h after the first one if the response is inadequate [10,107]. Some studies found that it is effective in severe COVID-19 cases [10,107]. Such therapy was suggested to decrease the risk of invasive mechanical ventilation or death in patients with severe COVID-19 pneumonia [108]. Several genetic variations have been demonstrated to modulate the efficacy of tocilizumab in RA, for example in *FCGR3A*, *IL6R*, *CD69*, and *GALNT18* genes, therefore, the same can apply to the treatment of COVID-19 patients [109,110,111]. However, no wide-scale pharmacogenomic studies of tocilizumab have been conducted in patients with any cytokine-release syndrome resembling the physiology in COVID-19. Some studies assessed the relationship between genetic polymorphisms within the *IL6R* gene and tocilizumab response [25]. It was found that carriers of IL6R rs4329505 CC and CT genotypes displayed worse response to tocilizumab compared with RA patients with TT genotype [112]. Furthermore, rs12083537 AA genotype correlated with reduced response to tocilizumab and increased risk for asthma when compared with the AG genotype. In turn, Maldonado et al. [110] found that the rs11265618 CC genotype was associated with enhanced response to tocilizumab in comparison to CT and TT genotypes. The presence of functional SNPs within the *IL6R* gene could exert an impact on the intracellular signaling pathway of the IL-6 receptor bound to tocilizumab [110]. Moreover, polymorphisms within the *FCGR3A* gene may modify the efficiency of treatment due to changes in systemic exposure. Patients with the *FCGR3A* rs396991 AA genotype may have an increased response to tocilizumab as compared with patients carrying AC or CC genotypes [109]. The study of patients with RA treated with tocilizumab demonstrated higher response at 12 months in carriers of *FCGR3A* rs396991 TT genotype (vs. GT; OR: 5.1; 95% CI: 1.2–21.3; *p* = 0.03) [109]. According to the authors, this variant may alter the affinity of the Fc fragment of IgG receptor to tocilizumab and change its systemic clearance. Greater response to tocilizumab was also reported in patients with the *CD69* rs11052877 AA compared to patients with the AG and GG genotypes, as well as in carriers of *GALNT18* rs4910008 CC individuals compared with patients with the CT and TT genotypes [111]. Variants in *CD69* and *GALNT18* genes were suggested to affect the downstream signaling pathways of the immune system in RA patients, therefore, their effects may not be visible in non-RA patients [111]. Currently, there is no strong evidence indicating that the analysis of pharmacogenomic biomarkers would be helpful in predicting response to tocilizumab therapy in patients with COVID-19.

## 9. The Renin-Angiotensin-Aldosterone System Inhibitors

The renin-angiotensin-aldosterone system (RAAS), composed of three main compounds: renin, angiotensin II, and aldosterone is a key regulator of blood volume and systemic vascular resistance [113]. The effects of ACE2 and ACE actions are antagonistic and therefore they provide a homeostatic regulation of angiotensin (Ang) II [25,114]. Renin participates in the transformation of angiotensinogen into Ang I, and in subsequent steps, Ang I is converted by ACE into Ang II (which induces vasoconstriction and hypertension via the interaction with angiotensin receptors 1R and 2R (AT1R, AT2R)), while ACE2 converts Ang II to Ang 1–7 exerting vasodilatory effects via the interaction with the Mas receptor. Eventually, Ang II promotes the release of aldosterone on the adrenal cortex, which controls blood volume, blood pressure, as well as levels of Na^+^, K^+^, and H^+^ [115,116,117]. As mentioned above, SARS-CoV2 can enter and infect the host pulmonary cells using the angiotensin-converting enzyme ACE2 receptor expressed in the membrane of pulmonary cells, therefore it has been suggested that ACE inhibitors and angiotensin-receptor blockers could negatively or positively interfere with the viral infection process [118,119]. Three types of drugs block the RAAS system acting at different levels: the inhibitors of the ACE (e.g., enalapril or lisinopril), the antagonists of the AT1R receptor (e.g., losartan or irbesartan), and, finally, the antagonists of aldosterone (e.g., spironolactone) [25]. The ACE inhibitors upregulate ACE2 receptors, thus they can potentially worsen the state of COVID-19 patients treated with ACE inhibitor therapy. Indeed, the upregulation of ACE2 was found to correlate with a worse COVID-19 prognosis [40]. In turn, at least in theory, angiotensin-receptor blockers could prevent virus entry at the cellular level [120]. Currently, several ongoing clinical trials are evaluating the effectiveness and safety of captopril (either alone or in combination) in patients with COVID-19 and severe pneumonia [10]. ACE inhibitors are among the most commonly used drugs in medical practice and, therefore, the pharmacogenetic aspects have been widely studied. The polymorphisms altering the disposition and response of RAAS inhibitors may affect the treatment of COVID-19 patients. For example, genetic variants of *CYP2C9* which metabolizes losartan may condition its clinical benefits [121]. The most frequent CYP2C9*2 and CYP2C9*3 variants are characterized by diminished function. The results of studies have demonstrated that two-thirds of the white population express the wild-type genotype (CYP2C9*1/*1), while one-third have either the *1/*2 or *1/*3 genotypes, and less than 2.5% of individuals possess the *2/*2, *2/*3, and *3/*3 genotypes [122]. *2 and *3 variants have been reported more frequently in patients with secondary kidney diseases and higher blood pressure compared with wild-type individuals [121]. Until now, no pharmacogenetic guidelines have been published concerning the adjustment of losartan doses based on *CYP2C9* genotyping, but such proceedings seem beneficial since disturbed drug metabolism could cause toxicity as a result of drug accumulation [25]. Moreover, the analysis of functional polymorphisms within the *ABCB1* gene could help to predict the response to losartan [123] since it encodes P-glycoprotein (P-gp) mediating the transfer of xenobiotics between body compartments, and the polymorphisms with its gene modulate drug pharmacokinetics. Variations rs1045642, rs2032582, and rs1128503 are among the most important polymorphisms in *ABCB1* [124]. In terms of ACE inhibitors, some studies have demonstrated the association between *ACE* rs1799752 and variability in the effectiveness of captopril, enalapril, and lisinopril [125,126]. Patients carrying del/del diplotype of this polymorphism (a 50-nucleotide deletion (del)) were found to show a worse clinical outcome. It appears that the determination of variation rs4961 located within the alpha-adducin gene enables the prediction of drug response in the case of the combination of spironolactone (aldosterone antagonist) and furosemide [127]. Individuals carrying the G allele showed a better response to treatment compared with T allele carriers. In turn, variability in COVID-19 prognosis has been suggested to be associated with polymorphism of the *ACE2* gene and the viral *ACE2* receptor gene [128]. Polymorphisms within the *ACE2* and *ACE* genes (*ACE2* rs4240157, rs4646155, rs4830542, rs2074192 rs233575, rs2158083, and rs21068809, *ACE* G8790A, and I/D) were found to correlate with hypertension, and since the hypertension influences COVID-19 prognosis, the analysis of ACE2 polymorphisms in this group of patients may be of interest [128]. Moreover, Sabater Molina et al. [129] found the relationship between four polymorphisms within the *ACE2* gene and COVID-19. According to their study, the presence of two SNPs: rs2074192 and rs1978124 appeared to exert a protective effect against COVID-19. They also showed that the DD genotype of *ACE1* in patients with comorbidities was associated with a greater risk of hospitalization due to COVID-19 and mortality. Similar results were obtained by Delanghe et al. [130] who demonstrated the relation between D-allele and increased COVID-19-associated mortality. The I/D polymorphism was found to affect *ACE2* protein levels in lung tissue which implies that it can modulate susceptibility to SARS-CoV-2 [131]. In turn, the occurrence of SNPs rs2106809 and rs2285666 correlated with a higher risk of hospitalization and severe course of the disease. Another study revealed the positive correlation between the alternate allele (T or A) of rs2285666 and the lower infection susceptibility and case-fatality rate in the Indian population [132]. Asselta et al. [133] suggested that the presence of the A allele might increase the strength of the splice site thus leading to higher ACE2 serum concentration. In contrast, Karakaş et al. [134] failed to demonstrate the impact of rs2106809 and rs2285666 on the severity of COVID-19 infection.

Until now, the results of studies failed to show any clinically significant effect of RAAS inhibitors in patients with COVID-19 [135]. There is also no evidence that ACE2 is upregulated in a dose-dependent manner in the course of such treatment, therefore, it seems that RAAS inhibitors should not be discontinued, especially bearing in mind that patients with COVID-19 and hypertension have a worse COVID-19 prognosis [136].

The summary of the pharmacogenetic impacts on the treatment efficacy and adverse effects is presented in Table 1.

## 10. Baricitinib

Recently, WHO has recommended two new medications for the treatment of COVID-19 [147]. One of them is baricitinib, an inhibitor of Janus kinase (JAK). Baricitinib received emergency use authorization from the US Food and Drug Administration for hospitalized patients with COVID-19 who required oxygen supplementation [148]. It is recommended for the therapy of patients with severe or critical COVID-19, in combination with corticosteroids, especially dexamethasone. However, the use of these two immunosuppressive drugs may enhance the risk of either opportunistic infections or the recurrence of latent infections [8]. Baricitinib’s mechanism of action involves the inhibition of immune system overstimulation. The treatment of hospitalized COVID-19 patients requires enhanced viral clearance and the limitation of the inflammatory response [149]. Baricitinib was demonstrated to hinder the signaling of the following cytokines: IL-2, IL-6, IL-10, IFN-γ, and G-CSF. The anti-inflammatory and anti-cytokine properties of baricitinib make it a promising drug that can be used in the treatment of patients with COVID-19 [150]. This drug also targets host proteins, including Numb-associated kinases (AAK1, BIKE, and GAK) which are responsible for SARS-CoV-2 viral propagation [149]. Before the pandemic, baricitinib was used to treat patients with rheumatoid arthritis. COV-BARRIER, a placebo-controlled clinical trial enrolling hospitalized patients with COVID-19 and increased level of at least one inflammatory biomarker demonstrated that the administration of baricitinib 4 mg orally had no significant effect on the occurrence of the primary endpoint (progression to high-flow oxygen), noninvasive ventilation, mechanical ventilation, or death by day 28 compared to placebo (OR 0.85; 95% CI, 0.67–1.08; *p* = 0.18) [150]. However, such therapy significantly diminished the mortality in the subgroup of patients receiving high-flow oxygen or non-invasive ventilation at baseline. Additionally, the results of ACTT-2 trial showed that a combination of baricitinib and remdesivir shorten the time to recovery in hospitalized patients with COVID-19 [151].

Baricitinib is metabolized by CYP3A4 [40,152]. Gene encoding this enzyme is considered as VIPs in the PharmGKB database. Therefore, the presence of *CYP2A4* gene variations can alter the safety or efficacy of baricitinib. It is the substrate of P-glycoprotein (Pgp), breast cancer resistance protein (BCRP), and multidrug and toxic extrusion protein (MATE) 2-K. The pharmacokinetics of baricitinib could be altered by Organic Anion Transporter 3 (OAT3) transporter encoded by the SLC22A8, thus it is not recommended in patients administered strong OAT3 inhibitors, including probenecid [40]. According to in vitro studies, baricitinib does not inhibit OAT1, OAT2, OAT3, organic cation transporter (OCT) 1, OCT2, organic anion transporting polypeptide (OATP)1B3, breast cancer resistance protein (BCRP), multidrug and toxin extrusion protein 1 (MATE1, also known as solute carrier family 47 member 1), and MATE2-K [153].

## 11. Molnupiravir

Molnupiravir is another new oral drug that received emergency use authorization (EUA) for the treatment of COVID-19 [154]. This drug can be used for the therapy of adults with mild to moderate symptoms of COVID-19 that appeared no more than 5 days earlier, in the case of patients who are at high risk of progressing to severe disease, and for whom alternative antiviral therapies cannot be used for any reason. Molnupiravir is administered in the form of a prodrug of N-hydroxycytidine (NHC). This small-molecule ribonucleoside displays the activity against SARS-CoV-2 and other RNA viruses [155,156]. The results of studies have demonstrated a high genetic barrier to NHC resistance [155]. Following the administration, circulating NHC is phosphorylated intracellularly to NHC triphosphate which becomes incorporated into viral RNA by viral RNA polymerase. Such incorporation leads to the accumulation of viral mutations and lethal mutagenesis. The occurrence of deleterious errors is associated with the incorporation of viral polymerase either guanosine or adenosine during viral replication which eventually render the virus noninfectious and unable to replicate [157,158,159].

The results of phase 1 and 2 clinical trials were promising therefore, this drug was included in the evaluation in the phase 3 trial [160,161]. The recommended oral dose of molnupiravir is 800 mg twice daily for 5 days, and only in the case where other therapies (remdesivir, ritonavir-boosted nirmatrelvir (Paxlovid), and sotrovimab) cannot be used.

The Phase 3 MOVe-OUT trial which enrolled non-hospitalized high-risk patients revealed that early initiation of treatment (within 5 days from the onset of symptoms) decreased the risk of hospitalization or any cause of death by 30% [154]. The efficacy of the therapy was found to be comparable in participants infected with the delta, gamma, and mu variants of SARS-CoV-2. However, in some groups of patients, including those with low baseline viral load, patients with evidence of previous SARS-CoV-2 infection, and those with diabetes mellitus, the outcomes were not significantly better compared to placebo [154]. The use of molnupiravir was found to be associated with mild adverse effects, including dizziness, nausea, and diarrhea. The results of in vitro studies have demonstrated that molnupiravir and its active metabolite NHC do not inhibit or stimulate any vital drug-metabolizing enzymes or inhibit drug transporters. Moreover, no significant drug-drug interactions have been observed yet.

## 12. Conclusions

Currently, no direct data concerning pharmacogenetics in patients with COVID-19 is available. The efforts of scientists have focused on finding effective treatment as fast as possible. The unraveling of mechanisms by which genetic determinants may modify the therapeutic outcome would enable the adjustment of treatment of COVID-19. Several genetic variants have been reported to modify the pharmacokinetics of hydroxychloroquine, ribavirin, azithromycin, lopinavir/ritonavir, tocilizumab, and others which hypothetically may influence clinical response and toxicity in the treatment of COVID-19. Currently, the level of evidence for most of them is still weak, and they have not been directly analyzed in patients with COVID-19, however, some of these potential pharmacogenetic relationships are worth further examination. One of the adverse effects which may be avoided by the adjustment of dose and drug based on pharmacogenetics is QT prolongation. Hydroxychloroquine, chloroquine, azithromycin, and lopinavir/ritonavir, as well as some combinations of drug–drug, drug–disease, and drug–gene interactions can individually increase the risk for QT prolongation and torsade de pointes in the course of COVID-19 treatment [162]. In the case of some drugs, the determination of pharmacogenetic markers has already been suggested (listed on the US FDA-recommended drug labels), including the G6PD gene for chloroquine and hydroxychloroquine and IFNL3 for ritonavir. The incorporation of pharmacogenetic knowledge in the prospective clinical trials of repurposed drugs used during COVID-19 therapy is essential to establish safe and effective dosing and to decrease the severity of adverse effects.

## Figures and Tables

**Table 1:** Impact of pharmacogenetic on the treatment efficacy and adverse effects.

Drug	Mechanism of Action	Pharmacogenomic Issues	Effectiveness of Treatment
Remdesivir	Inhibition of viral replication	CYP2C8, CYP2D6, and CYP3A4 (to a lesser extent) enzymes could have an impact on the metabolic conversion of remdesivir [27]. Polymorphisms within the *CYP3A4* gene may affect the response to the treatment [30].	4 randomized trials: Improved recovery and a slight decrease in mortality in adults with severe COVID-19. Diminished rate of serious adverse events [22]. Double-blind, randomized, placebo-controlled trial: remdesivir shortened the time to recovery in adults who were hospitalized with COVID-19 and had evidence of lower respiratory tract infection [24].
Lopinavir + ritonavir	Suppression of viral replication [2]	Lopinavir—rapid metabolism by the CYP3A4 enzyme—it should be co-administered with a low dose of ritonavir (CYP3A4 inhibitor) to reach optimal plasma concentrations [41,42]. Carriers of CYP3A4*22/*22 with or without *SLCO1B1* rs4149056—require a reduced dosage of lopinavir/ritonavir Carriers of rs28371759 CYP*18B within CYP3A4—faster metabolism of lopinavir [45]. Carriers of CYP3A5*3/*3 (lack of CYP3A5 expression)—require lower doses of lopinavir and ritonavir [45] rs2032582 alleles T and A, rs9282564 and rs2229109 in *ABCB1*—Increase the drug concentration rs8187710 in *ABCC2*—higher accumulation of lopinavir in peripheral blood mononuclear cells [48,49]. The rs4149056 in *SLCO1B1*—reduced OATP1B1 transport activity, variations of lopinavir plasma concentrations, and 37% lower clearance. Ritonavir increases concentrations of other medications metabolized by CYP3A4 [3]. Both drugs are contraindicated with drugs that are primarily metabolized by CYP3A enzymes or stimulate CYP3A [25]. Ritonavir may affect the biotransformation of some medicines metabolized via UGT-catalysed glucuronidation [3].	Systemic review demonstrated no significant clinical improvement compared to standard care with prominent adverse effect reactions [137]. Systematic review and meta-analysis (4 RCTs + 20 observational studies, 10,718 hospitalized COVID-19 patients)—lack of significant reduction of the risk of mortality, the decrease in the length of stay, the time for positive-to-negative conversion of SARS-CoV-2 nucleic acid tests [138]. No benefits associated with the reduction of the rate of mechanical ventilation. Significant 180% increase in the odds of adverse events [138]
Azithromycin	Inhibition of bacterial protein synthesis via binding to the 50S component of the 70S ribosomal subunit [54]	Pharmacokinetics can be influenced by the activity of P-gp transporter encoded by *ABCB1* [3,59]. Variations within *ABCB1* might enhance the treatment with azithromycin [57]. *ABCB1* rs2032582/rs1045642 genotype increases peak azithromycin concentrations in healthy volunteers after a single dose (500 mg). Higher systemic exposure to azithromycin (also in combination with hydroxychloroquine or chloroquine), may be even life-threatening (impact on QT prolongation—fatal arrhythmias) [40]	Treatment is associated with fewer interactions compared to other macrolides [58] Open-label non-randomized clinical trial: hydroxychloroquine and azithromycin should be administered to COVID-19 patients to cure the infection and limit the transmission of the virus [55] PRINCIPLE clinical trial indicated that azithromycin plus usual care did not markedly shorten the time to first self-reported recovery or decrease the risk of hospitalization—azithromycin should not be used routinely to treat COVID-19 in older adults unless there are additional indications [139] An open-label randomized clinical trial of patients in the UK found that azithromycin (500 mg once a day for 10 days plus usual care) did not improve the mortality at 28 days [140].
Chloroquine and Hydroxychloroquine	Inhibition of virus entry by targeting the endosomal pathway [17]. Inhibition of glycosylation of host receptors and proteolytic processing thus blocking viral entry [65]	rs762890235 and rs1316056737 variants in *ACE2* may prevent the interaction with the S protein and affect the clinical efficacy of HCQ or CQ [2] Hydroxychloroquine and chloroquine are substrates of OATP encoded by *SLCO14*-the variations within it may markedly affect the outcome of treatment [40]. Patients with G6PD deficiency should receive a reduced dose due to the risk of hemolysis, and hemolytic anaemia [77]. Both drugs are modest inhibitors of cytochrome P450 2D6, and inhibitors of P-glycoprotein—they may reduce the antiviral activity of remdesivir—coadministration is not recommended [141]	RECOVERY trial—HCQ did not decrease 28-day mortality compared with the usual standard of care. Patients treated with HCQ had a longer median hospital stay and were more likely to subsequently require intubation or die during hospitalization compared to those on standard care [142]. The COVID-19 Treatment Guidelines Panel does not recommend the use of chloroquine or hydroxychloroquine for the treatment of COVID-19 in hospitalized patients (AI) and in nonhospitalized patients (AIIa) [8]. Most severe complications: QTc prolongation and ventricular arrhythmias [80].
Corticosteroids	Suppression of cytokine release Inhibition of lung infiltration by neutrophils and other leukocytes [2]	Both responses to corticosteroids and drug-related toxicities may be associated with alleles variants in genes, including those involved in the receptor binding (e.g., *CRHR1*, *NR3C1*), metabolizing enzymes (e.g., *CYP3A4*, *CYP3A5*, *CYP3A7*, *GSTT1*), chaperone/cochaperone protein (e.g., *ST13*, *STIP1*, *FKBP5*) as well as transporters (e.g., *MDR1*, *ABCB1*) [90]. No pharmacogenetic recommendations have been made	Systemic use of corticosteroids for COVID-19 could decrease 28-day mortality in patients with critical and severe COVID-19 disease (RR 0.80, 95% CI 0.70–0.92) [88] Such therapy may increase the risk of mortality in patients with non-severe COVID-19. Systemic corticosteroids may diminish the need for invasive mechanical ventilation [88].
Atazanavir	Mediate in the inhibition of SARS-CoV-2 major protease [25,97,98].	Polymorphisms within *UGT1A1* (rs8175347, alleles UGT1A1*28, *36 and *37), the SNPs rs4148323 (UGT1A1*6) and rs887829 (UGT1A1*80)), *CYP3A4*, *CYP3A5*, and *SLCO3A1* genes may affect the efficacy of atazanavir [3,10,99]. Carriers of 1 or 2 copies of the *1 allele faster metabolism of atazanavir compared to those with one or more copies of the *3, *6, or *7 alleles [10,100]. Variants within *UGT1A1*, *UGT1A7*, *UGT1A3*, *APOE*, and *APOC3* genes—increased drug-related toxicity and ADRs (e.g., jaundice) [50,101,102]. Homozygous carriers of the UGT1A1*28 allele—increased risk of severe hyperbilirubinemia [103] Recommended counseling on the possibility of developing hyperbilirubinemia in carriers of UGT1A1*28/*28, *28/*37, *37/*37, and −364 TT [99]. *UGT1A1* genotyping in COVID-19 patients could improve therapy tolerability [3]. Homozygotic carriers of rs1045642 CC alleles may have higher plasma levels of this drug—increased risk of hyperbilirubinemia and severe jaundice. Carriers of *ABCA1* (rs542671182), *APOC3* (rs2070668, rs2854116, rs2854117), *APOA5* (rs 662799 and rs3135506), and *APOE* (ε2 and ε3 haplotypes)—increased the risk of dyslipidemia [105].	Atazavir blocks SARS-CoV-2 Mpro activity to a greater strength than lopinavir [97]. In Vero cells and a human pulmonary epithelial cell line it inhibited SARS-CoV-2 replication. In SARS-CoV-2-infected human primary monocytes and reduced IL-6 release. It reduced cellular mortality and cytokine storm-associated mediators. Clinical trials of atazanavir in combination with other drugs failed to demonstrate its efficiency in the treatment of COVID-19
Tocilizumab	Competitive inhibitor of IL-6-mediated signaling	*FCGR3A*, *IL6R*, *CD69*, and *GALNT18* may modulate drug efficacy [109,110,111]. No wide-scale pharmacogenomic studies of tocilizumab in COVID-19. Carriers of IL6R rs4329505 CC and CT genotypes—worse response to tocilizumab compared with TT genotype [112]. rs12083537 AA genotype—reduced response to tocilizumab and increased risk for asthma compared with AG genotype rs11265618 CC enhanced response to tocilizumab in comparison to CT and TT genotypes [110] *FCGR3A* rs396991 AA genotype—probably increased response to tocilizumab compared to AC or CC genotypes [109]. *CD69* rs11052877 AA and *GALNT18* rs4910008 CC—better response to tocilizumab compared to AG and GG genotypes and CT and TT genotypes, respectively [111].	Therapy could decrease the risk of invasive mechanical ventilation or death in patients with severe COVID-19 pneumonia [108].
The renin-angiotensin-aldosterone system inhibitors	Angiotensin-receptor blockers could prevent virus entry at the cellular level	CYP2C9*2, and CYP2C9*3 variants—diminished function—may affect clinical benefits and cause drug toxicity as a result of drug accumulation [25,122]. Functional polymorphisms within the *ABCB1* gene could help to predict the response to losartan [123]. rs1045642, rs2032582 and rs1128503—may alter drug pharmacokinetics [124]. *ACE* rs1799752—variability in the effectiveness of captopril, enalapril, and lisinopril [125,126]. *ACE* del/del diplotype—worse clinical outcome. Polymorphism of the *ACE2* gene and the viral *ACE2* receptor gene—variability in COVID-19 prognosis [128]. rs2074192 and rs1978124 in *ACE2*—protective effect against COVID-19 [129]. DD genotype of *ACE1* in patients with comorbidities—higher risk of hospitalization due to COVID-19 and mortality. SNPs rs2106809 and rs2285666—higher risk of hospitalization and severe course of the disease. Alternate allele (T or A) of rs2285666—lower infection susceptibility and case-fatality rate [132].	No association between the use of ACE inhibitors or ARBs and SARS-CoV-2 infection in the population of COVID-19 patients or patients with severe or fatal disease [143]. BRACE CORONA trial—no clinical benefit of cessation of ACE inhibitors or ARBs in patients hospitalized with COVID-19 [144]. Reduction in the odds of hospital admission in diabetic patients receiving RAAS inhibitors [135]. Long-term therapy with RAAS inhibitors—lower odds of admission to semi-intensive or intensive care compared to patients not treated with RAAS inhibitors [145]. Plausible mortality reduction in patients using ACE inhibitors/ARBs [146].

ARBs—Angiotensin receptor blockers; G6PD—glucose-6 phosphate dehydrogenase; HCQ—hydroxychloroquine; MHC—major histocompatibility complex.

## Data Availability

Not applicable.
